# Persistent enteric neuroinflammation chronically impairs colonic motility in a pyridostigmine bromide-induced mouse model of Gulf War illness

**DOI:** 10.1242/bio.061867

**Published:** 2025-06-06

**Authors:** Claudia A. Collier, Aelita Salikhova, Sufiyan Sabir, Shreya A. Raghavan

**Affiliations:** ^1^ Department of Biomedical Engineering Texas A&M University, 3120 TAMU, College Station, TX, 77843, USA

**Keywords:** Enteric neuroinflammation, Gastrointestinal motility, Gulf War illness, Neuro-immune, Pyridostigmine bromide, Enteric nervous system

## Abstract

Neuroplasticity in the adult colon enables the enteric nervous system (ENS) to adaptively remodel in response to acute inflammation, preserving motility. However, chronic inflammation may drive maladaptive neuroplasticity, resulting in gastrointestinal dysmotility, a hallmark of functional gastrointestinal disorders, including Gulf War illness (GWI). GWI affects ∼30% of Gulf War veterans and has been linked to oral toxic exposures during combat, such as pyridostigmine bromide (PB). To explore mechanisms of persistent dysmotility, we developed a PB exposure model relevant to GWI. In the colon, we observed structural and functional ENS changes, including an imbalance in excitatory and inhibitory motor neurons and altered motility patterns. These were accompanied by a sustained influx of pro-inflammatory macrophages and elevated cytokine levels, indicating persistent low-grade enteric neuroinflammation. Inflammatory macrophages were found near enteric neural stem cells (ENSCs), impairing their regenerative potential. Transcriptomic analyses corroborated the presence of chronic neuroinflammation and dysregulated repair pathways. Together, our findings suggest that persistent enteric neuroinflammation and impaired neurogenesis contribute to long-term colonic dysmotility in GWI. This model offers new insights into chronic ENS dysfunction and may guide therapeutic strategies for GWI and related disorders.

## Teaser

**Summary:** This study reveals how a one-time toxic exposure causes lasting gut nerve damage and inflammation, offering insight into chronic gastrointestinal symptoms seen in Gulf War veterans.

## INTRODUCTION

The gastrointestinal tract is a highly motile organ. Coordinated contractions and relaxations of gastrointestinal smooth muscle is responsible for digestion, nutrient absorption and waste elimination (Sarna, 2010). The colon is densely innervated by the enteric nervous system (ENS), whose cell bodies are housed entirely within the colon. Motor neurons of the ENS control and regulate motility (contractions and relaxations) of the smooth muscle intrinsically via neurotransmitters like acetylcholine and nitric oxide (Bornstein et al., 2004), among several others. Disruptions in colonic motility symptomatically manifest as chronic diarrhea/constipation and pain, significantly impairing an individual's quality of life (Black et al., 2020).

Importantly, the colon exists in a state of neuro-immune homeostasis, which adds a layer of regulation to its motility and function (Wang et al., 2022). Disruptions in neuro-immune homeostasis in the ENS due to inflammation impairs its ability to regulate colonic motility (Brierley and Linden, 2014). Inflammation in the colon may be secondary to infection, autoimmunity (like Crohn's Disease or other inflammatory bowel disorders), or toxic exposures (in the case of Gulf War illness; GWI) (Wogan et al., 2012; Padoan et al., 2023; Collier et al., 2024). In the short term, acute inflammation manifests as structural changes in the ENS resulting in neuroplasticity and disturbances in gut motility (Moynes et al., 2014; Sharkey and Kroese, 2001). In many cases, upon resolution of active inflammatory insult, a return to homeostasis occurs with repair and regeneration of the ENS, restoring functional colonic motility (Bercik et al., 2005; Spiller, 2004). However, persistent neuroplasticity and long-term impairment of colonic motility is observed in many functional gastrointestinal disorders including GWI, even in the absence of overt pathology (Brierley and Linden, 2014).

Functional gastrointestinal disorders, manifesting as chronic diarrhoea/constipation and abdominal pain is highly prevalent among Gulf War veterans experiencing GWI (Collier et al., 2024). While the onset of symptoms can be related to acute toxic exposures during combat in the Persian Gulf War 1990-1991, over 30% of Gulf War veterans continue to suffer from colonic dysmotility (Collier et al., 2024), defined broadly as abnormal or uncoordinated colonic motility, decades after the initial toxic insult was removed (Kates et al., 2022; Mawson and Croft, 2019; Fappiano and Baraniuk, 2020). The mechanisms behind the persistence of colonic motility disruptions in GWI are currently unknown.

The mode of toxic exposures in Gulf War were many (Elhaj and Reynolds, 2023; White et al., 2016): a central part of Gulf War toxic exposures involved the unregulated oral ingestion of pyridostigmine bromide (PB), to protect against potential nerve gas attacks (Charatan, 1999; Steele et al., 2015). We previously developed a mouse model, where oral exposure of mice to PB resulted in significant acute enteric neuro-inflammation, and disruptions in colonic motility (Collier et al., 2023). In our model, enteric neuro-inflammation manifested as an acute decrease in cholinergic motor neurons within the colonic myenteric plexus, a zone that houses the motor neurons of the ENS. Neuro-inflammation also manifested as a significant infiltration of macrophages into the muscularis externa (outer smooth muscle layers) and myenteric plexus. Macrophages within the muscularis externa and the myenteric plexus of the colon (i.e. away from the mucosa and the microbiome) bear a huge responsibility in integrating immune cues and maintaining neuro-immune homeostasis via their tolerogenic polarization, an anti-inflammatory immune phenotype that promotes tissue repair and limits excessive inflammation (Wood, 1975). Acute PB exposure in the mouse model demonstrated that tolerogenic polarization of muscularis macrophages was replaced by an inflammatory switch, where they produced high levels of inflammatory cytokines (Collier et al., 2023). However, in Gulf War veterans, neuro-inflammation is chronic and persistent even decades after toxic exposure was removed.

Therefore, in the current study, we established and characterized a mouse model of *chronic* gastrointestinal disruptions in GWI, where persistent colonic motility disruptions occur even when toxic and inflammatory insult is removed after short-term exposure. Using this model, we aimed to demonstrate mechanisms of persistent impairment in colonic motility to a one-time toxic exposure to PB, similar to those experienced by Gulf War veterans during combat. Inspired by animal models where acute inflammation was resolved by adult enteric neurogenesis aided by tissue-resident enteric neural stem cells (Spiller, 2004), we explored changes within the enteric neural stem cell compartment within the colon in GWI-related neuro-inflammation. In adults, enteric neural stem cells are multipotent progenitor cells that reside within myenteric plexi and self-renew and differentiate to repair and regenerate damage to the ENS (Bondurand et al., 2003; Estrada-Mondaca et al., 2007). Here, we tested that hypothesis that a single, short-term, acute inflammatory insult from PB exposure in the colon damages the enteric neural stem cell compartment, thereby preventing repair, regeneration and long-term recovery leading to persistent and chronic colonic dysmotility. Insight into mechanisms of persistence in inflammatory neuroplasticity may reveal new therapeutic or management strategies not just for Gulf War veterans, but also for a broader population that is afflicted with functional gastrointestinal disorders.

## RESULTS

### PB exposure persistently impacts enteric neural networks but not smooth muscle structures within the colon

**Fig. 1.**
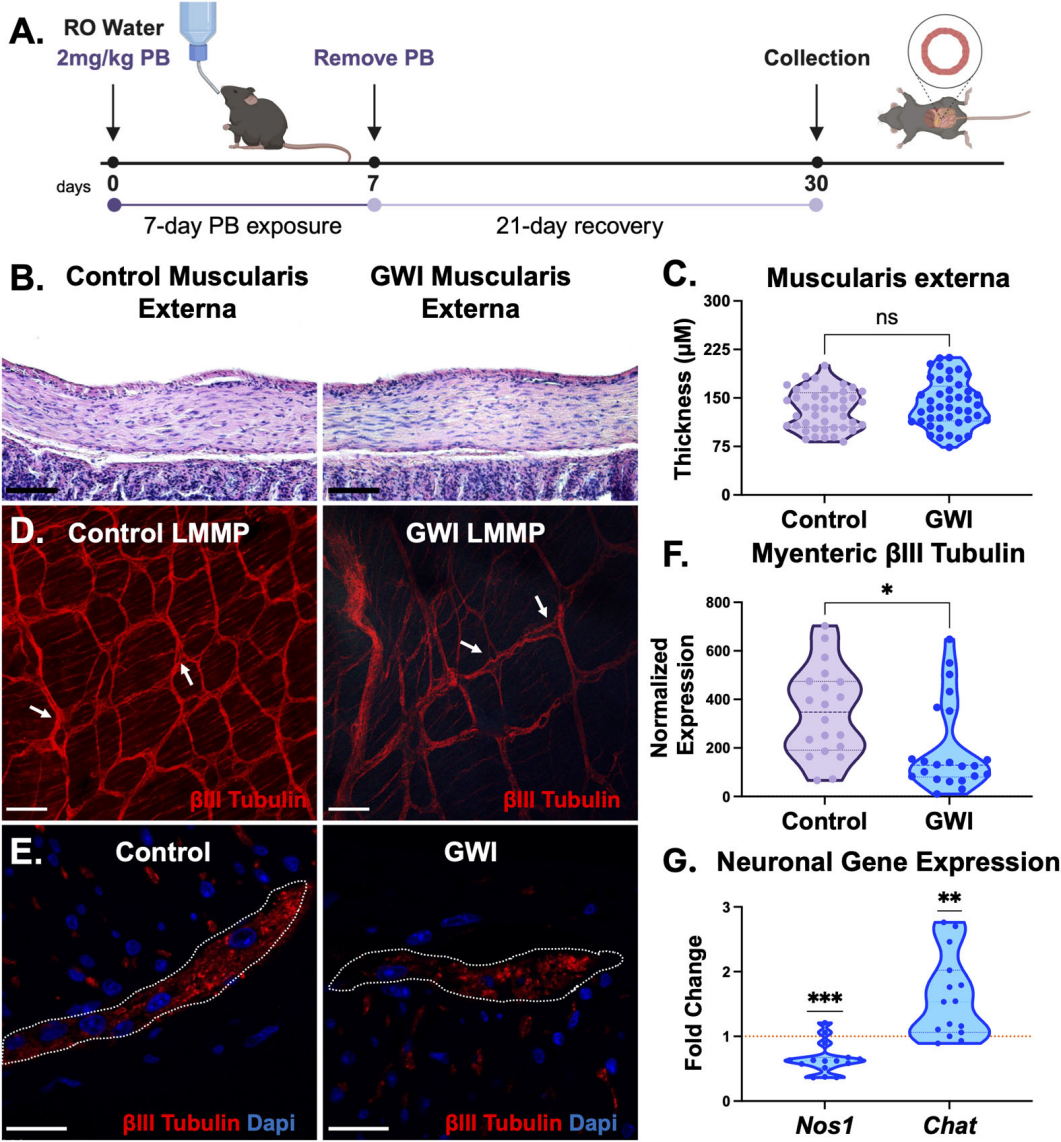
**Treatment regimen and effects of PB on the enteric nervous system and colonic musculature.** (A) Model of experimental timeline to assess the chronic (30 day) effects of a 7-day exposure of PB in mice. 32–34-week-old male mice were subjected to two random groups (control – given water; 7-day treatment of 2 mg/kg PB in drinking water) and after 7-day exposure, PB was removed from water and a 21-day recovery period was allotted to investigate late-onset of GWI-related symptoms. (B) Hematoxylin and Eosin (H&E) staining highlights cellular and structural morphology of colon cross sections from control and GWI mice (scale bar: 50 µm). (C) Quantification of smooth muscle thickness (from circular smooth muscle to longitudinal muscle) in H&E revealed no significant changes between control and GWI mice (ns, not significant, unpaired *t-*test, *n*=44). (D) Representative whole-mount preps of the longitudinal muscle and myenteric plexus (LMMP) were stained with pan neuronal marker, βIII-Tubulin, revealing the myenteric plexus network. Within control LMMP preps, ganglions contained bundles of dense nerve fiber tracks indicated by arrows. In GWI LMMP preps, ganglions are accompanied by gaps (shown with arrows) indicating a loss of a uniform neuronal network (scale bar: 90 µm). (E) Representative images of immunofluorescence staining of βIII-Tubulin on 20 µm colon cross-sections from control and GWI mice (scale bar: 25 µm). (F) βIII-Tubulin showed a significant decrease of expression within the myenteric plexus (*P<0.05, unpaired *t-*test, *n*=20). (G) Gene expression analysis of mature nitrergic neurons expressing *Nos1*, showed a significant decrease and cholinergic neurons (*Chat*) showed a significant increase (**P<0.01, ***P<0.001, one sample *t*-test, *n*=5). The dotted line at 1 represents the normalized level of gene expression in control colons. *n=*biological replicates.

To mimic patterns of PB exposure in the Gulf War, we administered PB orally to mice for 1 week and allowed a 3-week recovery period prior to any analysis of chronically persistent symptoms. This reflects the unregulated oral PB exposure observed in GWI, where individual variability was common (White et al., 2016). A schematic of this process is shown in Fig. 1A.

Smooth muscle structures of the colon are the effectors of colonic motility (i.e. the primary structures responsible for producing contractions and relaxation of the colon). Therefore, we first examined changes to overall smooth muscle structures. Standard Hematoxylin and Eosin staining was used to visualize the muscularis externa of the colon (the outer circular and longitudinal smooth muscle layers; Fig. 1B) in control untreated or PB treated mice. Following periods of recovery from one-time toxic PB exposure, no significant changes were observed in muscularis externa thickness (circular and longitudinal smooth muscle; compare 132.3±4.6 µm in control versus 141.1±5.53 µm in GWI, Fig. 1C).

In the absence of histological signs of smooth muscle hypertrophy, we undertook a broader characterization of the muscularis externa (the region broadly responsible for colonic motility). Motivated by our previous reports that oral PB exposure immediately induced acute enteric neuro-inflammation (Collier et al., 2023), we focused on enteric neural network integrity given its role in regulating colonic motility. Structural integrity of the enteric neural network was evaluated in whole mount preparations of the muscularis externa that retained the longitudinal muscle and the myenteric plexus of the enteric nervous system (LMMP). Staining with pan neuronal marker βIII-Tubulin demonstrated decreased neural fiber tracks including gaps (missing nerves indicated by arrows in Fig. 1D) in GWI colons, compared to fully intact control untreated colons. Additional visualization of βIII-Tubulin across the entire cross-section of the colon also demonstrated a significant and persistent loss in enteric neurons within the structures of the myenteric plexus (outlined in Fig. 1E). Quantification of βIII-Tubulin expression similarly denoted a significant 42% decrease in GWI colons compared to control colons (*P<0.05, Fig. 1F).

An orthogonal analysis at the gene expression level also demonstrated a significant 0.65-fold reduction in *Nos1* and 1.59-fold increase in *Chat* (Fig. 1G) in GWI colons, compared to control untreated colons.

Collectively, our data indicated that persistent effects from one-time PB exposure lingered via enteric neuronal impairment in form and function, but not smooth muscle. Therefore, we undertook a more thorough characterization of motor neuronal function in its ability to modulate smooth muscle contractility and relaxation, and, therefore, colonic motility.

### Chronic colonic dysmotility manifest in GWI colons due to neural impairment in the form of altered contractility and relaxation

**Fig. 2.**
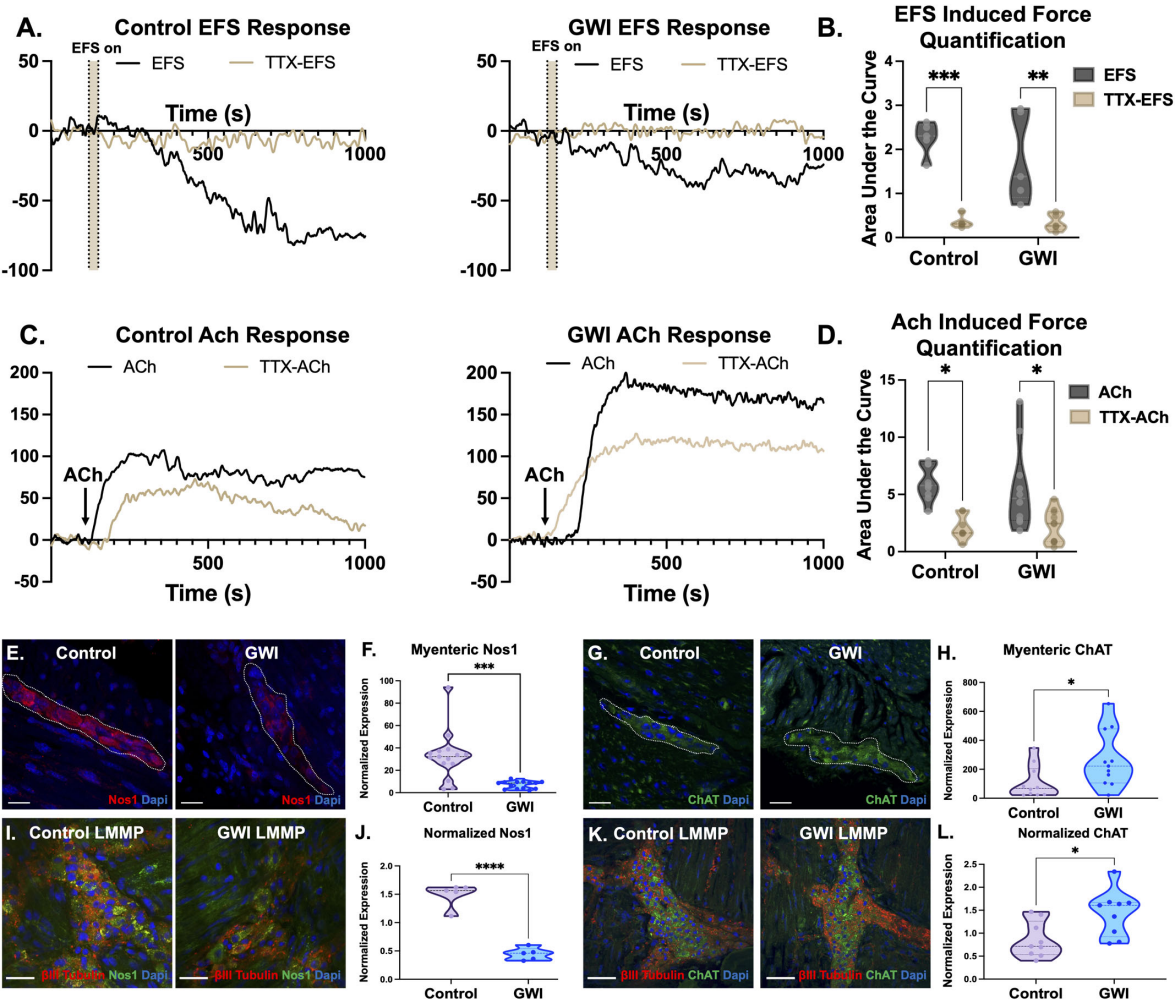
**GWI mice exhibit abnormal functional physiological responses of the motor neuronal and musculature component.** Organ bath measurements with a force transducer were used to evaluate force generation in colon explants through electrical field stimulation (EFS) (enteric nerve response) and exogenous Acetycholine (ACh) addition. (A) Force generation in response to EFS was measured in the presence of TTX (tan line) and absence (black line). The shaded region indicates the point in time of EFS activation (t=30 s) with parameters of 40 V, 10 Hz, 0.3 ms. Rapid responses to EFS were observed, where a relaxation of the colonic muscle resulted within control colon explants (black line) and in the presence of TTX, the electrically stimulated response was largely inhibited indicated by the steady force (tan line). The differences between the black and tan lines indicates robust enteric neuronal functionality, capable of mediating colonic relaxation in response to EFS. GWI colon explants decreased in force generation responses and was significantly reduced compared to control colon explants. In the presence of TTX, there is a significant decrease of electrically stimulated response. This indicates that the neural response in GWI colon explants is weaker and suggests neural impairment. (B) Graphical representation of the total area under the curve analysis for the uninhibited (EFS, black line) and inhibited groups (TTX-EFS, tan line) both in control and GWI colon explants. (**P<0.01, ***P<0.001, two-way ANOVA, *n*=5). (C) Force generation in response to exogenous ACh (1 µM) was measured in the presence of TTX (tan line) and absence (black line). Arrows indicate the point in time when ACh was added. ACh alone induced rapid contractions in control colon tissue (black line) while the presence of TTX inhibited the magnitude of the peak contraction, indicated by a decreased delta force (tan line). The changes in force due to TTX indicate the importance of a functional neuronal component in ACh contraction regulation. Within GWI mice, ACh alone (black line) demonstrated a significant increase in contraction compared to control mice. The higher magnitudes of force demonstrate the altered neuronal function, translating to signs of hypermotility. (D) Graphical representation of the total area under the curve analysis for the uninhibited (ACh, black line) and inhibited groups (TTX-ACh, tan line) both in control and GWI colon explants. (*P<0.05, two-way ANOVA, *n*=9). (E) Representative images of 20 µm cross-sections stained with nitritergic neuronal marker, Nos1, from control and GWI mice (scale bar: 25 µm). (F) Statistical analysis of the normalized expression focusing on the myenteric plexi (circled) showed significant decreased expression of Nos1 (***P<0.001, unpaired *t*-test, *n*=15). (G) Representative images of cholinergic neuronal marker, ChAT, from control and GWI mice (scale bar: 25 µm). (H) Statistical analysis of the normalized expression showed significant increased expression of ChAT (circled) (*P<0.05, unpaired *t*-test, *n*=10). Representative images of LMMP whole mounts from control and GWI mice colons co-stained with βIII-Tubulin and/or Nos1 and ChAT (scale bar: 20 µm). (I-J) The presence of nitrergic neuron expression, labeled with Nos1 (green), in GWI mice decreased significantly compared to control mice (****P<0.0001, unpaired *t*-test, *n*=5). (K-L) Cholinergic neuron expression, labeled with ChAT (green), within the myenteric plexus increased significantly in GWI mice (*P<0.05, unpaired *t*-test, *n*=9) *n*=biological replicates.

To evaluate whether enteric neural integrity and compositional changes in GWI altered colonic motility, we utilized an organ bath fitted with a force transducer to quantify colonic motility (Zakhem et al., 2014; Raghavan et al., 2011; Collier et al., 2023). Inhibitory enteric nerves within colon explants were stimulated using an electrical field from built-in platinum plate electrodes, following previously established protocols (Zakhem et al., 2014; Raghavan et al., 2011; Collier et al., 2023). Exogenous addition of the neurotransmitter Acetylcholine (ACh) was also used to evaluate both neuronally-evoked and myogenic contractions to a cholinergic stimulus. Fig. 2 shows representative force generation traces in response to either stimulation from control and GWI colon tissues, where the X axis follows time, and the Y-axis plots the magnitude of force generated from a stable baseline by the colonic explants.

In response to inhibitory electrical field stimulation (40 V, 10 Hz, 0.3 ms), control colons robustly relaxed, resulting in a reduced magnitude of force from baseline (black trace; Fig. 2A). The observed reduction in magnitude from baseline (relaxation) was indicative of being able to successfully stimulate inhibitory enteric nerves within the colon via electrical field (the tan band in Fig. 2A represents the duration that electrical field stimulation was on; t=30 s). To further confirm that the drop in force was a neuronally evoked response, electrical field stimulation was re-applied in the presence of a nerve blocker, Tetrodotoxin (TTX). Tan traces follow colonic motility response to electrical field stimulation in the presence of TTX. The lack of relaxation indicated the functionality of enteric nerves, and the specific ability of electrical field stimulation in evoking neuronally regulated responses resulting in colonic relaxation. Control colons averaged peak EFS-induced relaxation magnitudes of −89.92±0.99 µN, which was completely abolished in the presence of nerve blocker TTX (***P<0.001, Fig. 2A,B). In contrast, electrical field stimulation of GWI colon explants peaked much lower than controls, at −46.50±0.41 µN, including a similar abrogation of force in the presence of nerve blocker TTX (**P<0.01, Fig. 2A,B). The lower overall magnitude of relaxation in GWI colons was additionally indicative of inhibitory motor neuronal impairment translating to reduced functionality in its ability to mediate smooth muscle relaxation when stimulated.

To evaluate contraction, we used exogenous addition of the neurotransmitter Acetylcholine (ACh, its addition is indicated by the arrows in Fig. 2C). To delineate a neuronally evoked response from a myogenic contractile response to Acetylcholine, contractions were also measured in the presence of nerve blocker, TTX (tan traces; Fig. 2C,D). In response to exogenous addition of ACh, control colon explants contracted, averaging peak magnitudes at 77.50±4.6 µN (black trace; Fig. 2C). In contrast, average peak contractions in GWI colon explants were ∼37% higher, peaking at 110±0.9422 µN (quantified as area under the curve analysis in Fig. 2D). When enteric nerves were blocked via TTX pre-treatment, significantly reduced magnitudes of contraction were observed in both control and GWI colons (*P<0.05, Fig. 2D), indicating a functional cholinergic neuronal component capable of mediating smooth muscle contractions.

To better understand whether functional changes in motility aligned with underlying neuronal alterations, we next evaluated the expression of key inhibitory and excitatory motor neuron markers (Nos1 and ChAT) using both cross-sectional and LMMP staining. Evaluations of cross-sections for the expression of neuronal nitric oxide synthase (Nos1), a marker to denote the presence of inhibitory motor neurons that are capable of producing nitric oxide (Bornstein et al., 2004) demonstrated that within GWI colons, Nos1 expression significantly decreased by 80% compared to control colons (***P<0.001, Fig. 2E,F; myenteric plexi are outlined in the cross section). In contrast, quantification of choline acetyl transferase (ChAT; Fig. 2G) in cross-sections indicated a significant 2.40-fold increase in GWI colons compared to control colons (*P<0.05, Fig. 2H). We also visualized overall changes in these motor neuronal markers in LMMP preparations to get a more universal view within the myenteric plexus. Similar to cross sections, the presence of Nos1 (Fig. 1I,J) and ChAT (Fig. 1K,L) were evaluated in LMMP preparations. GWI LMMP preps showed a 3.3-fold decrease in nitrergic Nos1^+^ neurons compared to control LMMP (****P<0.0001, Fig. 1J). In contrast, cholinergic ChAT^+^ neurons showed a significant 1.68-fold increase in GWI LMMP compared to control LMMP (*P<0.05, Fig. 1L).

The combination of decreased relaxation responses (evoked by electrical field stimulation) and increased excitatory contraction (in response to exogenous addition of ACh) in GWI colons indicated a shift in neuronal balance within the enteric nervous system even after a one-time PB exposure.

### Chronic low-grade inflammation persisted in the muscularis externa of the GWI colon with skewed inflammatory macrophage polarization

**Fig. 3.**
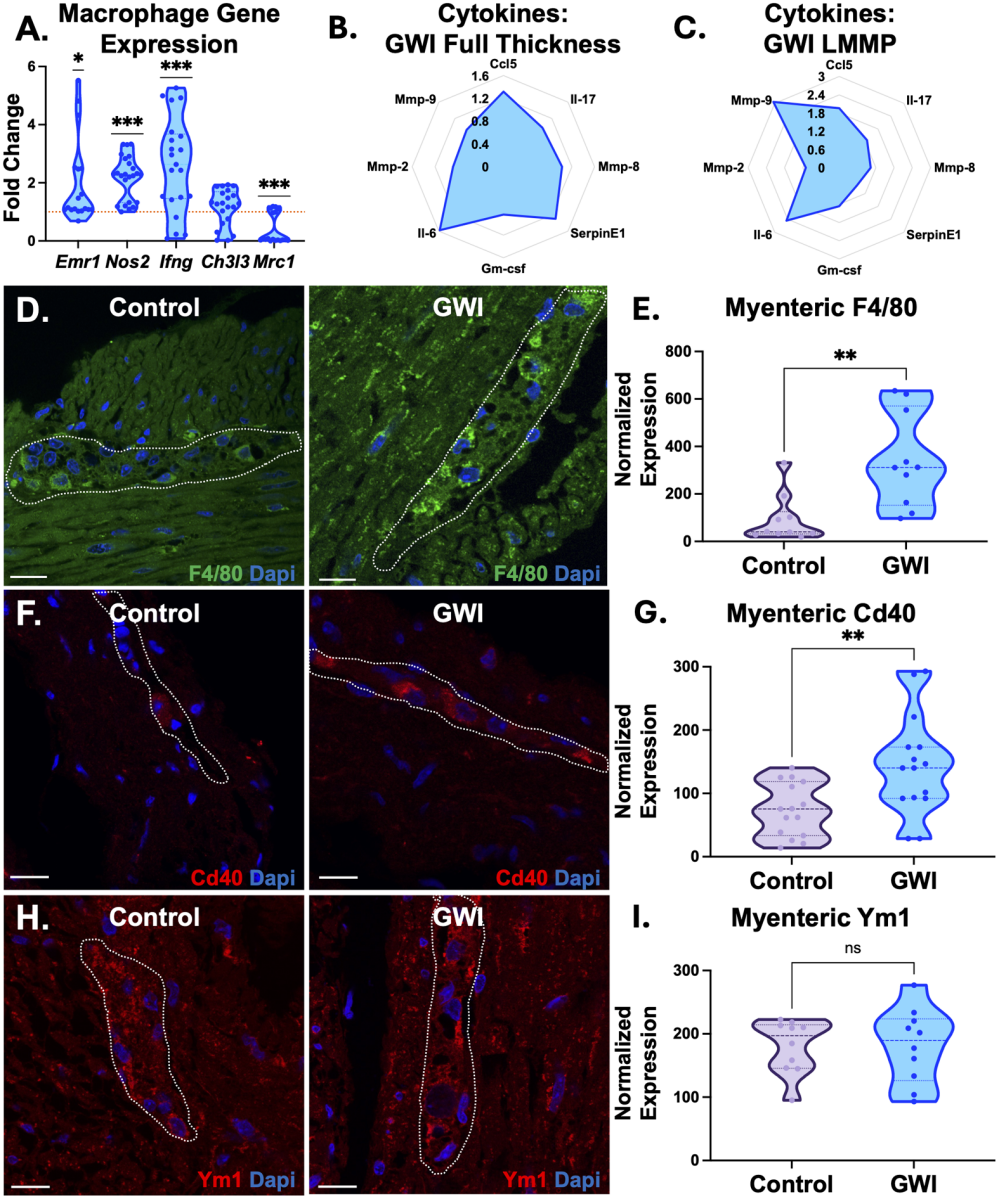
**GWI mice evaluated with a persistent low-grade inflammatory state especially within the myenteric plexus.** (A) Gene expression analysis of pan-macrophage marker (*Emr1*) and pro-inflammatory macrophage markers (*Nos2, Ifng*) showed significant increases (*P<0.05, ***P<0.001, one sample *t*-test, *n*=8); while pro-regenerative macrophage markers (*Mrc1*) showed significant decreases in expression compared to control colons. (***P<0.001, one sample *t*-test) The dotted line at 1 represents the normalized level of gene expression in control colons. (B-C) Radar plots of the average fold changes in cytokine levels from GWI mice used in a multiplex cytokine array are shown in two different layers: full thickness (encompassing the mucosa to serosa), and the LMMP. Il-6 cytokine levels were seen to be significantly higher in all layers of the colon. Other notable significances show that Mmp-9 was significantly increased within the LMMP layer in GWI mice. Immunofluorescent stains of macrophage markers – F4/80, pan-macrophage marker; Cd40, pro-inflammatory macrophage marker; Ym1, pro-regenerative macrophage marker, were used to evaluate the localization of macrophages within the myenteric plexi (circled) (scale bar: 25 µm). (D,E) F4/80 expression (green) was significantly increased within the myenteric plexi of GWI colons. (**P<0.01, unpaired *t*-test, *n*=10). (F,G) Expression of pro-inflammatory marker, Cd40 (red), also showed increased significance within GWI mice myenteric plexi (**P<0.01, unpaired *t*-test, *n*=15). (H,I) Both expressions of Ym1 (red) were similar throughout the myenteric plexus and muscularis externa. (ns, not significant, unpaired *t*-test, *n*=10). *n*=biological replicates.

Based on the persistence of gastrointestinal symptoms in Gulf War veterans (Collier et al., 2024), we hypothesized that even one-time PB exposure results in persistent and pervasive neuroinflammation, we examined the presence and polarization of a key inflammatory component of the muscularis externa, macrophages. Significant gene expression increases (*P<0.05, Fig. 3A) of pan-macrophage marker (*Emr1*), and pro-inflammatory macrophages (*Nos2* and *Ifng*) were observed in GWI colons compared to control, with no clear trends in anti-inflammatory gene expression between the tested markers.

Next, we validated low-grade inflammation via functional cytokine evaluation within control and GWI colons. Our analysis was split by full thickness samples, and just LMMP preparations to capture local inflammation within the muscularis externa, and the myenteric plexus. Cytokine profiles in Fig. 3B,C depict fold changes in samples from GWI mice, compared to control mice. Within full thickness colons (that comprise all layers including the epithelium), PB exposure resulted in a persistent significant increase in Il-6 (1.58-fold, ****P<0.0001, Fig. 3B), indicating a prolonged inflammatory state. Differences in other markers like Ccl5 and SerpinE1 were not significant between control and GWI mice. When evaluating cytokines within the LMMP layer alone, an elevated presence of Il-6 (2.5-fold, ***P<0.001, Fig. 3C) and Mmp-9 (3-fold, ****P<0.0001, Fig. 3C) were both observed in GWI mice, compared to controls. Even within the mucosa layer, increased Il-6 levels were sustained (Fig. S1). Overall, cytokine, chemokine, and secreted factor profiles indicated a sustained pro-inflammatory environment even after a long period of recovery from PB exposure in GWI colons.

To visualize the presence of macrophages within cross sections of control or GWI colon, a pan-macrophage marker F4/80 was used. On track with inflammation, F4/80^+^ macrophages were observed to be 3.75-fold higher in the myenteric plexi of GWI colons compared to control colons (**P<0.01, Fig. 3D,E). Myenteric plexi are outlined in the cross sections in Fig. 3. Once the significant presence of macrophages was established, macrophage polarization was evaluated as pro-inflammatory (Cd40) or anti-inflammatory (Ym1) states within the myenteric plexus. A significant 1.95-fold increase in myenteric Cd40^+^ macrophages was observed in GWI colons, compared to control (**P<0.01, Fig. 3F,G), with no significant differences in the presence of anti-inflammatory Ym1^+^ macrophages (ns, Fig. 3H,I).

### PB exposure chronically decreased the number of enteric neural stem cells in the colon

**Fig. 4.**
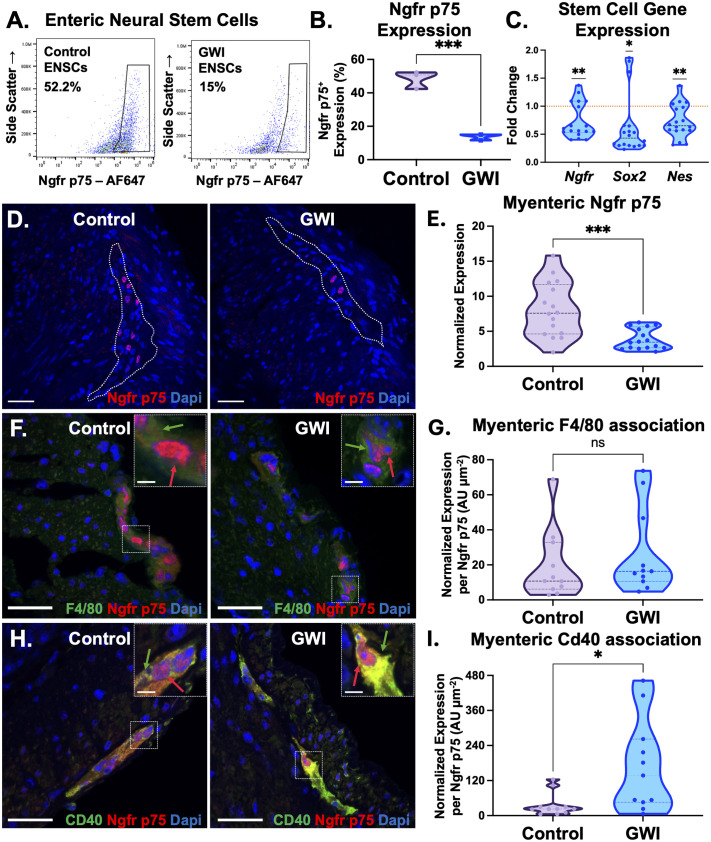
**Enteric neural stem cells decrease in GWI mice and are highly associated with inflammatory macrophages.** (A,B) ENSCs isolated from both control and GWI mice were evaluated for Ngfr P75^+^, an early detector marker for neural stem cells. Representative flow cytometry analysis plots showed a 0.28-fold decrease in the number of Ngfr p75^+^ enteric stem cells in GWI mice indicating an overall loss of ENSCs within treated mice (***P<0.001, unpaired *t*-test, *n*=3). (C) Gene expression analysis of other enteric neural stem cell markers (*Ngfr*, *Sox2*, *Nes*) showed significant decreases (*P<0.05, **P<0.01, one sample *t*-test, *n*=7). The dotted line at 1 represents the normalized level of gene expression in control colons. (D,E) Immunofluorescent staining with Ngfr p75^+^ on 20 µm colon sections show a significant decrease in the expression within the myenteric plexi (circled) (***P<0.001, unpaired *t*-test, *n*=16) (scale bar: 25 µm). (F,G) Immunofluorescent co-stains with F4/80, pan-macrophage marker, and Ngfr p75^+^ were evaluated to find associations between macrophages and ENSCs. Associations were determined by evaluating the corrected total fluorescence of F4/80 expression within a 20 µm radius of Ngfr p75^+^ expression. Both control and GWI mice exhibited similar associations within the myenteric plexi (ns, not significant, unpaired *t*-test, *n*=11), (scale bar: 25 µm). Inlet images show single Ngfr p75^+^ cell (red arrow) associated with F4/80^+^ macrophage (green arrow) (scale bar: 5 µm). (H,I) Associations were also done with Cd40, a pro-inflammatory macrophage marker, and Ngfr p75^+^. Quantification of Cd40 association to Ngfr p75^+^ showed a significant 4.72-fold increase of Cd40 association in GWI compared to mice (*P<0.05, unpaired *t*-test, *n*=11) (scale bar: 25 µm). Inset images show single Ngfr p75^+^ cell (red arrow) associated with Cd40^+^ macrophage (green arrow) (scale bar: 5 µm) *n*=biological replicates.

Our collective results so far demonstrated that a single, short-term exposure of the colon to pyridostigmine bromide perpetuated long-term, chronic enteric neural impairment and inflammation. Since p75^+^ enteric neural stem cells (ENSCs) are responsible for the regeneration and repair of myenteric nerves (Bondurand et al., 2003; Estrada-Mondaca et al., 2007), we evaluated broader changes to this cellular compartment upon PB exposure. Since these cells were rare to begin with, we enzymatically digested equal weights of colon from control or GWI animals, creating single cell suspensions amenable for flow cytometry analysis. Flow analysis revealed a significant ∼60% decrease (***P<0.001, Fig. 4A,B) in Ngfr p75 expression in GWI mice compared to control untreated mice. The gating strategy for all flow analysis is provided in Fig. S2. Orthogonally, a larger panel of genes related to ENSCs were also evaluated in control versus GWI colons, demonstrating significant decreases in *Ngfr p75*, *Sox2*, and *Nes* (*P<0.05, Fig. 4C). A significant 52% decrease of Ngfr p75 expression within the myenteric plexus (outlined) of GWI colons was also additionally evident by immunofluorescence staining, ***P<0.001, Fig. 4D,E).

### PB exposure increased the association of inflammatory macrophages with enteric neural stem cells in the colon

Given the impact of pyridostigmine bromide treatment on the number of Ngfr p75*^+^* enteric neural stem cells, we tested the hypothesis that chronic neuro-inflammation likely extended to the ENSC compartment of the colon. Cross sections of control or GWI colons were co-stained with F4/80, a pan-macrophage marker, and Ngfr p75, with representative images shown in Fig. 4F. Ratios of the total corrected fluorescence of F4/80 within a 20 µm distance of Ngfr p75 was quantified, revealing no significant differences between control and GWI mice (Fig. 4G). This indicated that a macrophage presence within the myenteric plexus, including near ENSCs was likely normal and unchanged with PB treatment chronically.

We then shifted our focus to evaluating the activation states of these macrophages in close proximity to ENSCs in control and GWI myenteric plexi. Specifically, we evaluated the inflammatory macrophage polarization marker Cd40 for its association with the ENSC marker p75 (Fig. 4H). A significant 4.66-fold increase of Cd40 inflammatory macrophages was observed with p75^+^ ENSCs in the GWI myenteric plexus, compared to controls (*P<0.05, Fig. 4I). Our data clearly indicated a chronic inflammatory immune association with ENSCs, implying an impaired ability to repair and regenerate mature enteric nerves that manifested in impaired colonic motility.

### Transcriptomic signatures revealed persistence of neuro-inflammatory and apoptotic pathways upon PB exposure in GWI colons

**Fig. 5.**
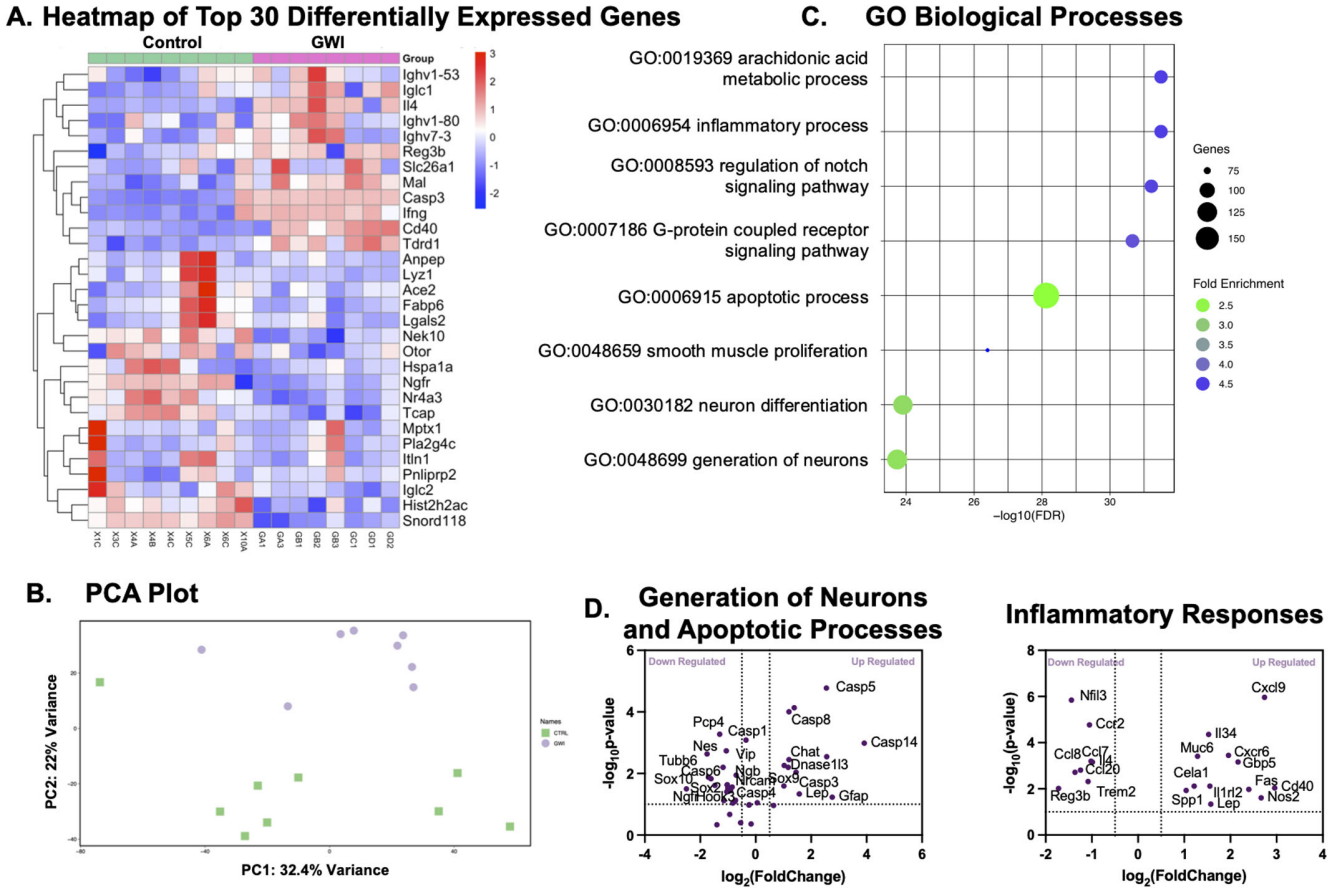
**Gene expression analysis of GWI and control mice.** (A) Heatmap of top 30 representative differentially expressed genes between GWI and control groups (>30% fold-change; FDR adjusted *P*<0.05). Expression values are scaled. Genes and samples are clustered by Euclidean distance. (B) PCA plot displaying all 17 samples along PC1 and PC2, which describe 32.4% and 22% of the variability, respectively, within the expression data set. (C) Bubble plot of representative BP enrichment results of differentially expressed genes. The bubble size reflects the number of genes enriched in a certain term, and the bubble color reflects the fold enrichment. (D) Volcano plots showing top up and down regulated genes within each biological process. FDR, false discovery rate; GO, gene ontology; BP, biological processes.

Unbiased RNA-Seq analysis was performed on colon tissues from control and GWI colons. 351 genes were uniquely downregulated and 135 were up regulated in GWI groups compared to the control group. Top 30 differentially expressed genes were plotted including up regulation of *Il4, Casp3, Cd40* and downregulation of *Ngfr, Nek10, Ace2, and Otor* (log2FC>±1.25 for all; Fig. 5A). Principal component analysis (PCA) revealed 32.4% variance in PC1, and 22% variance in PC2 (Fig. 5B), indicating broad differences between control and GWI colons due to one-time PB exposure, observed chronically despite a 21-day recovery period. Gene set enrichment analysis revealed immune and neural related pathways in both up and downregulated genes (Log2Foldchange=1.2; FDR value=0.1; Fig. 5C). This included notch signaling, apoptotic processes, generation, and differentiation of neurons. Pathways of smooth muscle proliferation and arachidonic acid metabolism of prostaglandins were also identified in the analysis.

To gain insights into these pathways, several biological processes (BP) pathways were broken down into their respective up- and downregulated genes within volcano plots. Within the generation of neurons pathway (Fig. 5D), the top three overexpressed genes were *Gfap, Chat*, and *Sox9* (log2FC>1.25 for all) in GWI colons, corroborating our previous results in cholinergic expression, not just from immunostaining but also in hypercontractile phenotypes. The top five under expressed genes included *Ngfr, Nes, Tubb6, Sox2*, and *Shh* (log2FC>−1.25 for all), indicative of impaired neural regeneration from ENSCs. Genes *Casp14*, *Gsdmd*, *Casp5*, and *Lep* were upregulated in GWI mice related to the apoptotic process (log2FC>1.5 for all; Fig. 5D).

Lastly, within inflammatory responses pathway, the top expressed genes were *Cd40*, *Cxcl9*, *Nos2*, *Fas*, and *Gbp5* (log2FC>2 for all) within GWI mice (Fig. 5D), further corroborating the presence of inflammation within the colon and the myenteric plexis.

## DISCUSSION

Neuroplasticity is the ability for adaptive remodeling of neural circuits in response to injury or inflammation (Calderone et al., 2024). It is essential for maintaining homeostatic gastrointestinal function and motility. However, in the context of chronic inflammation, neuroplasticity can become maladaptive, leading to sustained structural changes in the ENS ultimately disrupting gut motility and function (Demir et al., 2013). In the context of GWI, over 30% of Gulf War veterans continue to suffer from chronic diarrhea/constipation and abdominal pain (severe functional gastrointestinal motility disorders), related to acute toxic exposures that occurred ∼20 years ago, during the Persian Gulf War (Collier et al., 2024). Why Gulf War veterans continue to experience gastrointestinal symptoms is a significant knowledge gap, which we aimed to approach from the lens of persistent enteric neuro-inflammation and maladaptive neuroplasticity within the colon.

In previous work, we developed a mouse model that demonstrated acute colonic motility disruptions upon oral exposure to PB for 7 days (Collier et al., 2023). Among the many toxic exposures during combat, the only oral toxic exposure was the unmitigated ingestion of PB as a prophylactic measure against nerve gas attacks (White et al., 2016). Since our focus was on understanding colonic motility disruptions, we continued to build upon our oral PB exposure model, adding a 3-week recovery period from PB exposure to evaluate chronic effects. Our findings of damaged enteric neural structures (Fig. 1) are in alignment with reports of sustained structural changes in the enteric nervous system in the context of chronic inflammation (Demir et al., 2013), including in irritable bowel syndrome, diverticular disease, and in aging (Deduchovas et al., 2008; Hanani et al., 2004; Törnblom et al., 2002).

Several other groups adopt oral PB exposure to induce gastrointestinal symptoms associated with GWI, reporting alterations in gastrointestinal motility via bead expulsion assays (Chaudhari et al., 2024) and direct recordings of contractility *ex vivo* (Hernandez et al., 2019). We similarly adopted the use of *ex vivo* contractility recordings from isolated circular structures of the colon, drawing from our expertise in evaluating colonic motility using these analytical methods (Zakhem et al., 2014; Raghavan et al., 2009, 2011, 2014). Therefore, using an isometric force transducer, we quantified changes in colonic motility in response to exogenously added acetylcholine or direct electrical field stimulation of the enteric nerves within control untreated or PB-treated and recovered colons. Similar to data reported by Hernandez et al. (2019), despite a 3-week recovery following one-time PB exposure, persistent colonic dysmotility (hypercontraction) was observed in response to cholinergic excitatory stimulation (Fig. 2). Interestingly, while our previous acute model showed reduced cholinergic contraction (Collier et al., 2023), here, we observe increased excitatory contractile responses alongside reduced inhibitory relaxation, indicating a neuroplasticity-driven shift in the balance of excitatory and inhibitory signaling. The reversal in findings between acute and chronic settings implied neuroplasticity mechanisms at play, contributing to impaired repair and regeneration. Indeed, chronically, an imbalance was identified between excitatory and inhibitory neurons via immunofluorescence (Fig. 2), specifically the loss of inhibitory nitrergic Nos1^+^ neurons. The losses we observed in the GWI colons have also similarly been observed in animal models of diarrhea-prone irritable bowel syndrome and type 1 diabetes (Li et al., 2016; Masaoka et al., 2014; Camilleri et al., 2011; Takahashi et al., 1997; Wrzos et al., 1997). Collectively, our findings suggested maladaptive neuroplasticity in GWI. Therefore, we investigated enteric neuro-inflammation as a likely cause in the chronic GWI colon.

An inflammatory soluble milieu and an influx of inflammatory macrophages was observed in the GWI colon myenteric plexus (Fig. 3). Muscularis macrophages, the main immune cell within the muscularis externa, are particularly important for maintaining immune homeostasis (De Schepper et al., 2018). When these otherwise tolerogenic macrophages acquire pro-inflammatory programming (Mikkelsen et al., 2008), broader inflammation is instigated within the gut as observed in aging, post-operative ileus (Tsuchida et al., 2011; Farro et al., 2017; Yuan and Taché, 2017), diabetic gastroparesis (Grover et al., 2017; Bernard et al., 2014; Grover et al., 2018, 2019) – all conditions also accompanied by disrupted colonic motility (Becker et al., 2018). In our chronic GWI model, despite inflammation being chronic and persistent, we classify it as low-grade based on the cytokine release profiles (Petersen and Pedersen, 2005). While this chronic low-grade inflammation may not affect the structural smooth muscle component, it can significantly disrupt colonic motility (Peuhkuri et al., 2010), reported in the contexts of inflammatory bowel disorders (Raouf et al., 2024; Zhang et al., 2024; Kinoshita et al., 2006; Bercík et al., 2004; Melgar et al., 2005; Robinson et al., 2017; Vanheel et al., 2014), obesity and diabetes (Zogg et al., 2023; Antonioli et al., 2020, 2019; Cruz-Muñoz et al., 2024), and diverticulitis (Tursi, 2019; Scaioli et al., 2016; Cossais et al., 2019). Interestingly, in a wildly different context of airway smooth muscle, chronic persistent low-grade inflammation in asthma results in smooth muscle hypercontractility (Berair et al., 2013; Akiho et al., 2002; An et al., 2007). We believe that our observations of hypercontractility (in response to ACh) in GWI colons may be a similar response to chronic low-grade inflammation. Thus, the heightened contractile response may represent a downstream effect of chronic, unresolved inflammation and aberrant neuroplasticity, rather than a purely myogenic adaptation. This interplay between persistent inflammation, neural remodeling, and motility dysfunction supports the hypothesis that maladaptive enteric neuroplasticity is a driver of GWI-related dysmotility.

Having confirmed a low-grade inflammatory milieu in the GWI colon and muscularis externa, we continued to investigate whether this specifically impacted neuroplasticity via repair and regeneration of the enteric nervous system. To the best of our knowledge, the focus on impaired long-term repair and regeneration within the enteric nervous system in GWI symptom persistence is novel. Disruption of the gut-brain axis through gut microbiome dysbiosis and inflammatory gliosis are more frequently studied and documented as indirect causal factors in persistent gastrointestinal dysmotility in GWI (Chaudhari et al., 2024; Hernandez et al., 2020, 2019). However, the ENS functions largely independently of the central nervous system in regulating gastrointestinal motor activity and motility (Schneider et al., 2019), and houses its own tissue-resident ENSCs that contribute to its repair and regeneration. p75^+^ ENSCs, therefore, are key cellular players responsible for repairing damaged neural networks restoring colonic function to homeostasis (Burns et al., 2004; Burns and Thapar, 2014), by promoting neurogenesis and neuroplasticity (Kulkarni et al., 2017).

The close proximity of inflammatory Cd40^+^ macrophages with ENSCs (Fig. 4) suggests local amplification of inflammatory cytokines, that may potentially impact ENSC ability to repair and regenerate the enteric nervous system. Our results are aligned with other reports where enteric neuroinflammation (even instances of resolved inflammation) cause critical long-term alterations to neuroplasticity (Masson et al., 1996; Lin et al., 2005), resulting in chronic motility dysfunction. Our results therefore underscore that proximity of local enteric neuroinflammation impacts the ability of ENSCs to repair and regenerate the enteric nervous system, disrupting motility long-term in GWI. This was additionally corroborated even with an unbiased approach via pro-inflammatory and apoptotic pathways upregulated at the transcriptome level (Fig. 5).

Unlike transient inflammatory responses, where homeostasis is restored once the inflammatory insult is resolved, GWI represents a unique case in which acute, one-time exposure to PB resulted in lasting disruptions to neuro-immune structure and function. This persistent neuroplasticity in response to inflammation could explain why symptoms of diarrhea, constipation and abdominal pain continue to affect GWI veterans decades after the initial exposure. Similar neuroplasticity-inflammation driven dysmotility has been documented in other models of gastrointestinal dysfunction, particularly in functional gastrointestinal disorders (FGIDs), where chronic neuroinflammation impairs the ENS’s capacity for repair and regeneration (Mawe, 2015; Spear and Mawe, 2019; Lakhan and Kirchgessner, 2010). In these conditions, sustained inflammation and neural remodeling prevent the re-establishment of neuro-immune homeostasis, perpetuating motility issues. The overlap between GWI-related dysmotility and FGID mechanisms suggests that GWI could serve as a model for understanding the broader impacts of neuroplasticity and inflammation on chronic gut dysfunction. By advancing our understanding of cellular dynamics in enteric neuroinflammation, we can pave the way toward effective, targeted treatments for chronic gastrointestinal motility disorders.

## MATERIALS AND METHODS

### Materials and reagents

All tissue culture media, and supplements were purchased from ThermoFisher Scientific (Waltham, MA, USA), unless otherwise specified. Collagenase type II was purchased from Worthington (Lakewood, NJ, USA) and dispase from StemCell Technologies (Canada). B27 supplement was purchased from ThermoFisher Scientific. Growth factors were purchased from Peprotech (Cranbury, NJ, USA). All conjugated antibodies were purchased from Santa Cruz (Dallas, TX, USA) or Abcam (Cambridge, MA, USA), unless specified otherwise. Pyridostigmine bromide (PB) was purchased from Sigma-Aldrich (St. Louis, MO, USA). Acetylcholine and tetrodotoxin was purchased from Sigma-Aldrich.

### Chronic effects of PB: mouse model of GWI

Expanding on our previously established acute GWI-related model of oral PB exposure leading to enteric neuroinflammation (Collier et al., 2023), we adapted our model to investigate the chronic effects of PB exposure. C57BL/6 male mice (32-34 weeks old), purchased from Jackson Laboratories (Bar Harbor, ME, USA), were chosen to model middle-aged physiology, reflective of the current demographic of Gulf War veterans, and to capture age-related vulnerability in neuro-immune repair mechanisms. Briefly, after male mice were acclimatized, PB was administered through oral exposure of 2 mg/kg through drinking water for 7 days. Average water intake was estimated at 4-6 ml/day/mouse, in line with previous oral dosing models (Bachmanov et al., 2002). After 7 days of PB exposure, PB was removed from the water and mice were maintained with access to regular water until euthanization at 30 days. This ‘recovery’ period allowed us to investigate chronic effects even after the acute toxic exposure was removed. All experimental protocols were approved by the Texas A&M University Animal Care and Use Committee (IACUC AUP 2020-0182 D).

### Immunofluorescence methods

#### Colon whole-mount longitudinal muscle and myenteric plexus (LMMP) preparation

Mice were euthanized by carbon dioxide euthanasia followed by cervical dislocation. Whole-mount LMMP sections were prepared through previous established methods (Collier et al., 2023). Briefly, colon sections were dissected, and fecal content cleaned with sterile Hanks’ Balanced Salt solution (HBSS). The colon was cut longitudinally as to open and expose the mucosa layer, then the mucosa and submucosal layers were gently peeled off leaving behind the longitudinal muscle and myenteric plexus (LMMP). Tissue sections were immediately placed in neutral buffered formalin for 4-6 h at 4°C.

#### Paraffin slide preparation and whole-mount immunohistochemistry

Paraformaldehyde-fixed samples were processed at 4 µm and stained with Hematoxylin and Eosin, performed by the Texas A&M University veterinary medicine and biomedical sciences research histology unit core facility. Paraffin-embedded colon tissues from control and GWI mice were sectioned into 20μm sections. Paraffin sections were rehydrated following deparaffinization followed by antigen retrieval performed by means of 1× sodium citrate buffer (pH 6.0) for 20 min in 100°C water bath. Whole-mount immunohistochemistry (IHC) protocols were adapted from Levin et al. (2021) and previously detailed IHC protocols (Collier et al., 2023). Briefly, LMMP sections cured overnight in 50% glycerol, then placed in 30% sucrose with an overnight incubation at 4°C. After permeabilization and blocking for 3 h, each tissue was subsequently stained with fluorophore-conjugated antibodies (Table S1) and an additional nuclear counterstain, Dapi. A detailed list of antibodies used is included in the supplementary materials. Unbound antibody was washed with PBS, and tissues were mounted using Prolong Diamond anti-fade reagent (ThermoFisher Scientific) before imaging using a Leica SP8 confocal microscope at the Microscopy and Imaging Center at Texas A&M University.

### Gene expression via qPCR

RNA was extracted from explant control and GWI colon using a RNeasy extraction kit (Qiagen, Hilden, Germany). RNA concentrations were measured using a Nanodrop 2000 (ThermoFisher Scientific) spectrophotometer, transcribed to cDNA using the high-fidelity cDNA transcription kit (Life Technologies), and qPCR was carried out in the 96-well format using a QuantStudio (Applied Biosystems). Gene expression differences were quantified using the 2ΔΔC_T_ method, using Gapdh as the housekeeping control, and reported as fold changes compared to a control age-matched sample. qPCR experiments were run in triplicates. Primer sets used are detailed in Table 1.

**Table 1.** List of primers used in RT-qPCR analysis of gene expression

Mouse primer	Gene sequences
*Gapdh*	F: AGGTCGGTGTGAACGGATTTG R: GGGGTCGTTGATGGCAACA
*Nos1*	F: CCCAACGTCATTTCTGTCCGT R: TCTACCAGGGGCCGATCATT
*Chat*	F: GGCCATTGTGAAGCGGTTTG R: GCCAGGCGGTTGTTTAGATACA
*Ngfr*	F: TGCCGATGCTCCTATGGCTA R: CTGGGCACTCTTCACACACTG
*Sox2*	F: GCGGAGTGGAAACTTTTGTCC R: GGGAAGCGTGTACTTATCCTTCT
*Sox9*	F: AGTACCCGCATCTGCACAAC R: ACGAAGGGTCTCTTCTCGCT
*Nes*	F: CCCACCTATGTCTGAGGCTC R: GGGCTAAGGAGGTTGGATCAT
*Emr1*	F: CTGCACCTGTAAACGAGGCTT R: GCAGACTGATGAGTTAGGACCACAA
*Nos2*	F: GGAGTGACGGCAAACATGACT R: TCGATGCACAACTGGGTCAAC
*Mrc1*	F: CTCTGTTCAGCTATTGGACGC R: TGGCACTCCCAAACATAATTTGA
*Ifng*	F: ACAGCAAGGCGAAAAAGGATG R: TGGTGGACCACTCGGATGA

### Cytokine multi-plex assay

Colon samples were collected from control and GWI mice. Tissue was processed for multiplex immunoassay as previously described (Collier et al., 2023). Briefly colon tissues were separated into three groups: sections containing all layers of the colon (full thickness), the longitudinal muscle and myenteric plexus (LMMP), and the mucosa. Tissues were homogenized and lysed in cell lysis buffer 2 (R&D systems, Minneapolis, MN). Protein concentration was measured using a Pierce BCA assay (ThermoFisher Scientific). Cytokine levels (Table 2) were evaluated using bead-based multiplex immunoassays and done according to the manufacturer's protocol (R&D Systems, Minneapolis, MN, USA).

**Table 2.** List of cytokines used in bead-based multiplex immunoassay

Cytokine
C-C Motif Chemokine Ligand 5 (Ccl5)
Interleukin-2 (Il-2)
Interleukin-6 (Il-6)
Interleukin-17 (Il-17)
Serine protease inhibitor clade E member 1 (SerpinE1)
Granulocyte macrophage-colony stimulating factor (Gm-csf)
Matrix Metalloproteinase-2 (Mmp-2)
Matrix Metalloproteinase-8 (Mmp-8)
Matrix Metalloproteinase-9 (Mmp-9)

### Measurement of physiological functionality of mice colon tissue rings

Physiological testing protocols were adapted from previous work from Collier et al. with detailed description of this protocol (Collier et al., 2023). Briefly, a custom-built organ bath set up was used, with a force transducer (FT20 from Harvard Apparatus, Holliston, MA, USA). One end of explanted colon rings was secured to the fixed pin within the organ bath, while the other end was looped over the measuring arm of a force transducer. The organ bath was filled with 37°C DMEM+HEPES. The force transducer was calibrated, and noise readings from air and warm bath fluid were established prior to mounting colonic tissues. DMEM+HEPES immersing the colon ring was changed every 30 min, or at the end of experimental acquisition, whichever was earlier.

A 20% stretch was applied to colon rings using a micromanipulator, maintaining this length for the experiment duration to establish a new equilibrium (60-80 min). The baseline force at 20% stretch was arbitrarily set to zero to observe the effects of added neurotransmitters or electrical field stimulation. Testing protocols assessed overall colon physiology and enteric neuronal contribution to force generation by using Tetrodotoxin (TTX; 1 μM) as a nerve blocker. Comparisons were then made between control and GWI colon constructs. Colon constructs were treated with 1 μMol/ml acetylcholine (ACh) to study force generation through neurotransmitter-induced receptor-mediated cholinergic response. Colon constructs were also treated with electrical field stimulation (EFS) (40 V, 10 Hz, 0.3 ms) to study relaxation in baseline in response to neurotransmitters.

### Isolation and culture murine enteric neural stem cells

Enteric neural stem cells (ENSCs) were isolated from colon samples from control and GWI mice as previously described (Collier et al., 2023). Briefly, colon tissues underwent several incubations of a collagenase and dispase digestion mix, adapted from Raghavan et al. (2013). Isolated cells were plated in neuronal growth media (Neurobasal+1× B27 supplement+1× antibiotics+10 ng/ml EGF+10 ng/ml bFGF). ENSCs were maintained in culture, with growth media supplementation every 2 days. Cells were passaged by collection, manual pipetting for disruption of spherical aggregates, and replated in fresh growth medium every 5 days.

### Flow cytometry

ENSCs were characterized using flow cytometry for NGFR p75 expression (Metzger et al., 2009), by adapting previously used flow analysis protocols (Raghavan et al., 2019; Collier et al., 2023). Briefly, ENSCs were collected and resuspended in a flow cytometry amenable buffer (PBS+2% FCS) at a concentration of 50,000 cells/ml. Cells were incubated with a p75-AF647 antibody, with a matched isotype control. Cells were then processed through a flow cytometer (Attune NxT, Waltham, MA, USA). Gates were established based on the isotype control cut off at 0.5% (see Fig. S2).

### RNA Isolation for RNA-sequencing

Colon explant tissue samples from both control and GWI mice were flash frozen using liquid nitrogen and then lysed and homogenized in TRIzol reagent (Invitrogen, Carlsbad, CA, USA). RNA was then extracted using a RNeasy extraction kit (Qiagen, Hilden, Germany). RNA concentrations were measured using a Nanodrop 2000 (ThermoFisher Scientific) spectrophotometer. Library preparation with poly(A) selection and 150-bp paired-end sequencing on an Illumina HiSeq 2500 were performed by Azenta (South Plainfield, NJ, USA).

### Computational analysis of RNA-seq data

Raw data files in FASTQ format were generated from the Illumina sequencer. To examine the sequencing quality, the quality score plot of each sample was plotted. After quality control, sequence reads were trimmed to remove possible adapter sequences and nucleotides with poor quality using Trimmomatic v.0.36. The trimmed reads were mapped to the Mus musculus GRCm38 reference genome available on ENSEMBL using the STAR aligner v.2.5.2b. Unique gene hit counts were calculated by using featureCounts from the Subread package v.1.5.2. After extraction of gene hit counts, the gene hit counts table was used for downstream differential expression analysis. Using DESeq2 (R package), a comparison of gene expression between the control and GWI defined groups of samples was performed. The Wald test was used to generate *P*-values and log2 fold changes. Genes with a *P*-value <0.05 and absolute log2 fold change >1 were called as differentially expressed genes for each comparison. PCA analysis was performed using all differentially expressed genes. This analysis aimed to compare overall transcriptional profiles between groups after a 3-week recovery period following PB exposure. Heatmap and k-means clustering were performed to visualize the expressed genes using iDEP tool suit. Expressed genes were used to create Volcano plots using GraphPad Prism v.10.1.1 (264). The threshold for *P* value cutoff of the expressed gene was assigned ≤0.05. WGCNA was performed in R using genes with a minimum module size of 30 to isolate relatively large gene modules and detection in at least 10% of samples were included in the analysis. To best capture patterns of co-regulation, a signed network was used. Using the pickSoftThreshold function, we empirically determined a soft threshold of 14 to best fit the network structure. Gene ontology (GO) annotations of biological process (BP) were performed EnRichGO using R library. We filtered original pathways analyzed with *P* values <0.05 and false discovery rate <0.05.

### Statistics

All statistical analysis was performed on GraphPad Prism v.10.1.1 (264) (GraphPad Software, San Diego, CA, USA; www.graphpad.com) and RNA-seq analysis was done in R (v.2024.09.0+375). All reported values are means±s.e.m., resulting from 5-20 biological replicates, depending on the experiment. IHC quantification was calculated based on corrected fluorescence intensity measured through ImageJ, highlighting the myenteric plexus and subtracting the area times background with 10-15 biological replicates (Bankhead, 2014). This method was used for all fluorescent quantifications including βIII-Tubulin, Nos1, ChAT, F4/80, and Ngfr p75. Normalized expression of LMMP Nos1 and LMMP Chat was calculated based on the corrected total fluorescence intensity over the corrected total fluorescence of βIII-Tubulin expression. IHC macrophage association to Ngfr p75 was calculated based on the macrophage corrected total fluorescence within a set distance (20 µm) of Ngfr p75 expression. Flow analysis differences were shown for potential significance in GraphPad Prism v.10.1.1 with three biological replicates. H&E muscularis externa mean size were calculated through ImageJ (v.2.14.0t) with 44 biological replicates. Force transducer data was acquired by Power Lab Data Acquisition System and LabChart8 (Colorado Springs, CO, USA) at a frequency of 10 Hz. GraphPad Prism 10.1.1 was used for all further data analysis. Second order Savitsky-Golay smoothing was applied to raw data. Values were exposed as the mean and standard error of the mean of 8-12 experiments. EFS-induced relaxation and ACh-induced contraction were expressed as absolute changes in force from baseline in micronewtons (μN). Seven tests were used for testing significance. All qPCR data was normalized to control conditions within each experimental set and performed in triplicates over at least five biological replicates. ELISA data was normalized according to protein concentration of analyzed samples by dividing all interpolated concentration results by the concentration of protein in each well then showcased as fold-changes and graphed through Excel (v.16.79.1). The interpolation was performed according to a 5-term logarithmic interpolation using a standard curve created per manufacturer instructions. Five biological replicates were used for testing significance. ANOVA-based hypothesis testing, and unpaired *t*-tests were used where appropriate. Statistical significance (defined as *P*≤0.05) were indicated within each experimental data set, with associated *P*-values.

## Supplementary Material

10.1242/biolopen.061867_sup1 Supplementary information
