# Supplementary material for: Persistent enteric neuroinflammation chronically impairs colonic motility in a pyridostigmine bromide-induced mouse model of Gulf War illness
Source: Biol Open. 2025 Jun 6;14(6):bio061867. doi: 10.1242/bio.061867 (PMC12171577; doi:10.1242/bio.061867)
Supplement: Supplementary information [file biolopen-14-061867-s1.pdf]

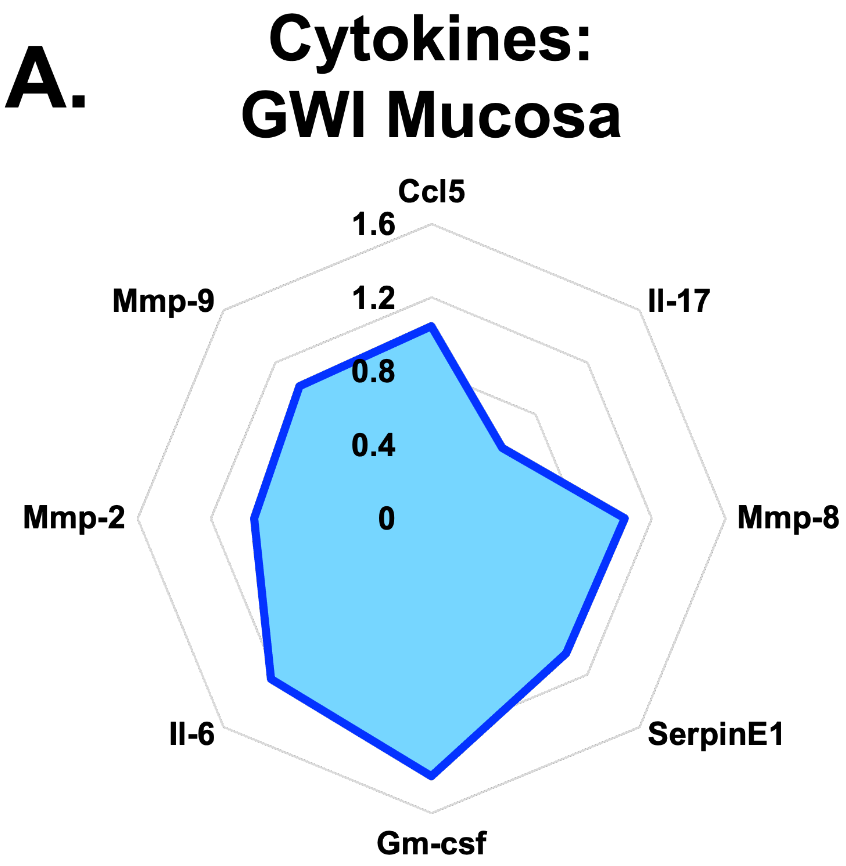

Fig. S1. Inflammatory cytokine profiles consistent within the mucosa layer.
(A) Radar plot of the average fold changes in cytokine levels from GWI mice used in a multiplex cytokine array specially within the mucosa layer. Assessing the cytokine profile within the mucosa layer, there were no significant changes in most markers between control mice and GWI mice except for Il-6, which exhibited a 1.23-fold increase (***p<0.001).

Flow Cytometry Gating Technique

Flow cytometry analysis was completed with FlowJo (Ashland, OR). Polygon gates were drawn on isotype flow cytometry graphs to establish a background gate at 0.5%. The same gate was applied to antibody-stained cells to establish a percent positive population.

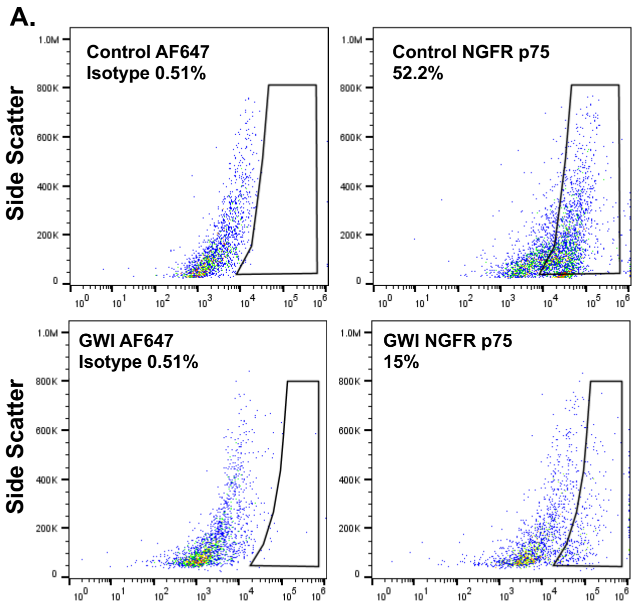

Fig. S2. NGFR p75 flow cytometry gating technique using isotype controls. (A) Polygon gate (outlined in black) was drawn on FlowJo to create a 0.5% background cut off gate in the isotype control graph (left). The same gate was applied to the antibody-stained graph (right) to establish the percentage of the population expressing NGFR p75.

Table S1. List of antibodies and dilutions used in immunohistochemistry.

Antibody	Conjugated Fluorophore	Dilution	Manufacturer	Application Used	Catalog #	Batch #	RRID
βIII-Tubulin	Alexa Fluor 647	1:100	Santa Cruz Biotechnology	IHC	sc-80005	A2119	AB_2210816
ChAT	FITC	1:50	Santa Cruz Biotechnology	IHC	sc-55557	B2222	AB_2291743
F4/80	Alexa Fluor 488	1:100	Abcam	IHC	ab204266	1010237-1	AB_2943479
Ym1	PE	1:100	Abcam	IHC	ab211621	1078389-1	AB_3083045
Cd40	Alexa Fluor 680	1:50	Santa Cruz Biotechnology	IHC	sc-514493	L2821	AB_3678831
Ngfr p75	Alexa Fluor 647	1:50	Santa Cruz Biotechnology	F, IHC	sc-271708	J1816	AB_10714958

Biology Open • Supplementary information
